# A research protocol on leap motion tracking device: A novel intervention method in distal radial fracture rehabilitation

**DOI:** 10.1371/journal.pone.0267549

**Published:** 2022-05-06

**Authors:** Sakshi P. Arora, Waqar M. Naqvi

**Affiliations:** 1 Department of Community Health Physiotherapy, Ravi Nair Physiotherapy College, Datta Meghe Institute of Medical Sciences, Wardha, Maharashtra, India; 2 Directorate of Research, NKP Salve Institute of Medical Sciences and Research Centre, Nagpur, Maharashtra, India; Prince Sattam Bin Abdulaziz University, College of Applied Medical Sciences, SAUDI ARABIA

## Abstract

**Introduction:**

Physiotherapeutic rehabilitation are used to optimize functional recovery following a distal radial fracture (DRF). Being most common upper limb fracture in all age groups, the DRF peaks in young men and in post-menopausal women with incidence ratio of 1:4. Leap motion control based rehabilitation of patients with DRF is limited. This research aims to assess the efficacy of leap motion control based rehabilitation in patients with DRF.

**Methods:**

In an randomized parallel group trial, subjects (n = 40) with DRF will be recruited. The participants will be enrolled into either experimental or control group with 1:1 allocation ratio. Following the primary assessment and allocation, the participants in experimental group will receive both leap motion control and conventional therapy over a period of six weeks. Participants in conventional group would undergo only conventional therapy. The primary outcome measures will be Disabilities of the Arm, Shoulder, and Hand (DASH) questionnaire and Universal goniometer however the grip strength and Visual Analog Scale (VAS) will be used as secondary outcome measures.

**Purpose of the study:**

The findings of this trial will examine the impact of leap motion control in DRF patients with conventional therapy on improving the functional activity, range of motion (ROM), grip strength and pain.

**Expected clinical implications:**

To conclude, this research seeks to examine the rapid and long term effects of leap motion control in DRF patients. The study findings would help prospective patients with DRF, which may include a newly designed approach of rehabilitation.

## Introduction

Distal radius fractures (DRF) is a frequent site of injury in upper extremity fracture and are amongst the mostly encountered fractures in emergency rooms [1, 2]. There is a considerable association of DRF on functional activities of patient affecting the socioeconomic costs and deteriorating standard of living [3, 4]. Being most common upper limb fracture in all age groups with bimodal distribution, the DRF peaks in young men and in post-menopausal women with incidence ratio of 1:4 [4–6].

In younger people, DRF is mostly associated with fall associated during sports events and road traffic accidents [7]. The incidence of DRF in females is significantly greater than in males incorporating the menopause as responsible factor leading to osteoporosis associated with reduced bone density showing peak between the age group of 60 and 70 [8].

In older adults, the preferred line of management is conservative or non-operative to have good results with foundation stone of immobilisation [9]. Following DRF, many factors manipulate the recovery of the patient implying the age, gender, site and extent of injury, management line followed to manage the respective injury, compensations, patient’s education regarding the condition, radial shortening, and intra-articular involvement [10]. Patients generally recover within 3–6 months to maximum range of motion, strength and function whether managed conservatively or surgically [7].

Patients with DRF after a duration of immobilisation are often referred for physiotherapy. In the clinical setting physiotherapists use range of motion (ROM) and grip strength to determine progress as well as outcomes. Hand activity requires a combination of adequate sensation, proprioception, intact neurological control and coordination, appropriate anatomical alignment, and muscle strength and flexibility [8]. Physical therapy (PT) interventions are techniques used to improve functional recovery following a DRF [10–12].

The introduction of virtual reality (VR) technology into traditional training has the ability to further increase the outcomes of the training. VR allows users to actively interact in real-time with a simulated environment and offers the opportunity to practice skills learned in the virtual environments to everyday life [13]. VR-based training has the ability to promote implicit learning, improve variety, and involve the patient actively during the training. Such characteristics are crucial in the optimization of motor learning and could maximize the training impact in DRF patients.

The Leap Motion Controller is an infrared light detector developed as a means of hand gesture recognition. The corresponding software applies algorithms to the sensor data detected from the hands and generates a 3D representation of contour, position and movement. Current applications include gaming, education, maps and navigating the computer desktop. The technology lends itself to monitoring hand movement exercises used for wrist physiotherapy. However, no current research exists that investigates the feasibility and validity of using this technology. The aim is to assess the impact of leap motion control based rehabilitation on hand function and functional independence after distal radius fracture.

## Material and methodology

### Ethical approval

The study proposal was ethically approved by Departmental Research Committee, Ravi Nair Physiotherapy College on 31^st^ March, 2021 with reference number DMIMS(DU)/IEC/2021/235. It was registered in the Clinical Trial Registry-India (CTRI) with the registration number CTRI/2021/05/033498. The preprint of this protocol is presently available with digital object identifier (doi) https://doi.org/10.21203/rs.3.pex-1340/v1 in the repository named The Protocol Exchange [14].

### Study design

**Fig 1:**
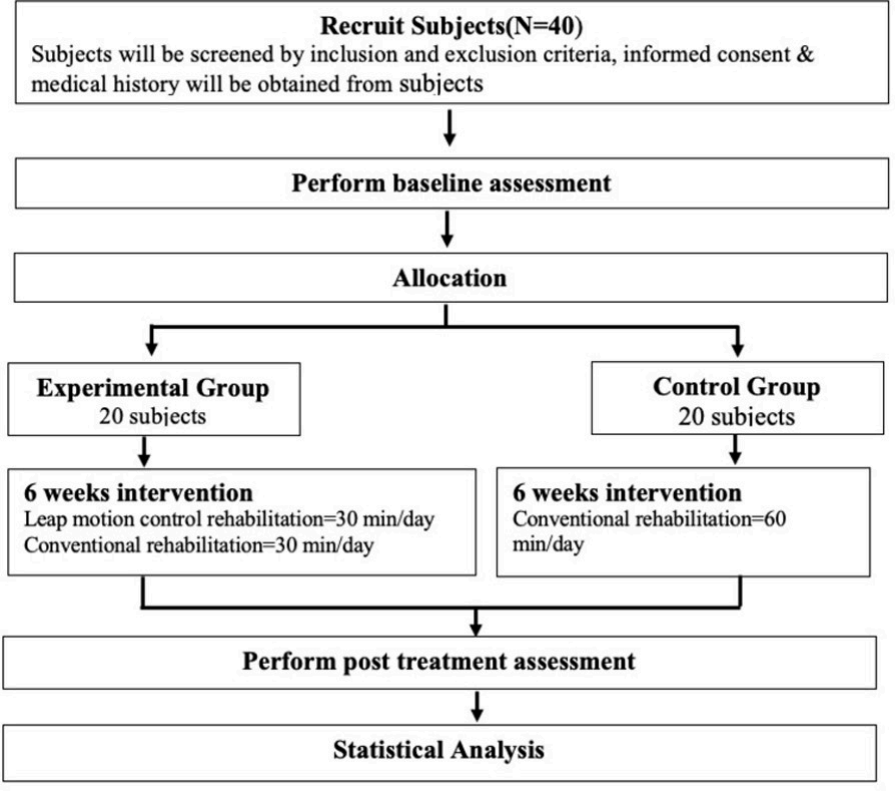
Study flow-chart. Step-wise procedural plan of recruitment, allocation, intervention, and statistical analysis of the study.

This randomized parallel group trial will be carried out in the HumEn Research Lab of Ravi Nair Physiotherapy College, Sawangi (Meghe), Wardha, after approval from Institutional Ethics Committee of Datta Meghe Institute of Medical Sciences, Deemed to be University and Clinical Trial Registry-India. Before inclusion, all the participants will be informed regarding the aim and procedure of research. Those participants who will meet the inclusion criteria must give the written informed consent. In an experimental study, those participants (N = 40) diagnosed with DRF will be enrolled for six weeks protocol. Fig 1 shows the flow chart of the study.

### Trial design

It is a randomized parallel group trial in which the patients will be allocated into two independent groups. Group A will be control group getting the conventional physiotherapy rehabilitation program while Group B will be assigned with experimental group. The method that will be used for generating random sequence will be computer generated randomisation. The concealment will be done by sequentially numbered, sealed, opaque envelopes method and the participant will be blinded in the study.

### Participants

The inclusion criteria includes 18–45 years age patient with DRF conservatively managed in a cast or managed with the k-wires under a cast, no previous history of wrist/ hand fracture, history of inflammatory arthritis, or any possible upper limb fracture and ability to recognize and obey basic verbal instructions [15].

The exclusion criteria includes patients with bilateral fracture, patients with past trauma either in arms or hands that had impaired function, rheumatic and neurological disorder patients, malignant conditions and symptomatic osteoarthritis of wrist and hand.

### Sample size consideration

**Fig 2:**
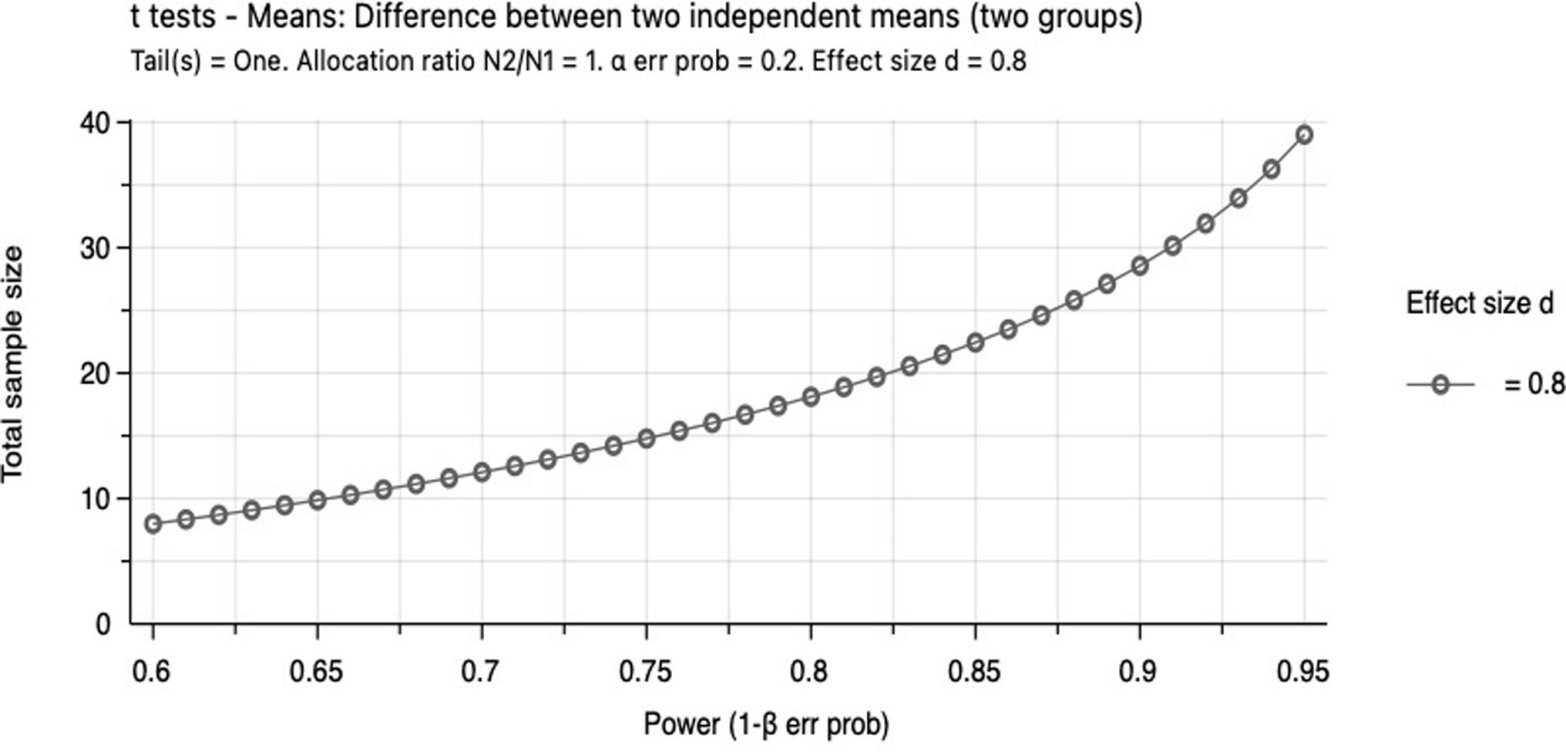
The calculation of sample size using G*Power analysis. Graphical representation of the distribution of power on X-axis versus total sample size on Y-axis.

This is an experimental study with two-groups design that examine the effect of leap motion control in distal radius fracture rehabilitation. Analysis of previous power using G*Power is used to determine the sample size [16]. 40 participants will be enrolled to the control group or experimental group and will be allocated accordingly [12]. The G*Power analysis followed the following terms for calculation of sample size which is graphical represented in Fig 2.

**t tests**—Means: Difference between two independent means (two groups)
**Analysis:** A priori: Compute required sample size
**Input:**   Tail(s)      =   One
     Effect size d     =   0.8
     α err prob     =   0.2
     Power (1-β err prob)      =   0.95
     Allocation ratio N2/N1    =   1
**Output:**   Non-centrality parameter δ    =   2.5298221
     Critical t    =   0.8511828
     Df       =   38
     Sample size group 1    =   20
     Sample size group 2    =   20
     Total sample size      =   40
     Actual power        =   0.9531573

### Intervention design

Group A: Those subjects in control group should undergo conventional training for 60 minutes a day, five days a week for six weeks. This group will receive conventional physiotherapy program consisting of Whirlpool, Joint mobilisation (Maitland, Kalternborn grade-1 gliding, grip strength exercises, scapular retraction exercises, Home Exercise Program (HEP). Some of the most common therapeutic approaches used to optimize the patient’s functional recovery are physical pain management strategies such as TENS, heat therapy. The range of motion (ROM) exercises would include passive mobilization of forearm and wrist, active exercises and active-assisted exercises for forearm, wrist, and fingers [7]. The PT strengthening plan includes exercises to strengthen wrist and finger flexors and extensors, forearm supinators and pronators (against therapist’s manual resistance and/ or elastic bands), and gripping activities (using several soft balls). Conventional therapy will be structured to replicate the skills needed in the leap motion environment with comparable intensity and complexity. Study researchers would encourage and supervise participants to ensure the consistency of the study.

Group B: Those subjects in experimental group with the Leap motion control will be asked to actively move their fingers from initial resting position and execute maximally the following five movements with their wrist and hand: fingers flexion and extension, flexion and extension of the thumb, wrist radial and ulnar deviation, forearm pronation and supination and wrist flexion and extension [3] for 30 minutes regularly. Rehabilitation games include-

1. Rhythm game: This game is aimed at helping patients perform flexion exercises for wrist joint. The buttons fall from above as music plays in the background, and must be pushed at the right time by the player.
2. Flappy Bird Clone: It is designed for wrist flexion and extension both in which the player is asked to control a bird flying over a series of tubes before the track ends.
3. Skiing Game: It requires wrist movement i.e. either extension or flexion and wrist radial or ulnar deviation. A skier who comes down a slalom track is guided by the player and must cross the gates [17].

### Outcome measures

Primary outcome measure:

1. Disabilities of the Arm, Shoulder, and Hand Outcome Questionnaire (DASH): It is a 30- item questionnaire that evaluates the patient’s ability to perform upper extremity activities for functionality [18].
2. Universal Goniometer for ROM: Active flexion and extension of the wrist will be measured by a Standard BASELINE® 12-inch plastic goniometer, (Model 12–1000) Fabrication Enterprises, Inc: White Plains, New York. Participants will be assessed to expose their arms in a seated position and to remove any accessories [19].

Secondary outcome measure:

1. Grip strength: To check grip strength a Jamar (5030J1) Hydraulic dynamometer by JLW Instruments, Chicago, will be used. The participants would be tested in sitting position, with their arm placed to the side of the body; their shoulders in neutral position, elbow in 90° of flexion, and forearm in neutral [19]. Then, subjects will be asked with all their strength to make tight fists to maintain that position for four seconds and then rest for 30 seconds. First, the unaffected side will be tested and then affected side will be tested. The maximum value obtained from 3 trials will be recorded.
2. VAS: The visual analogue scale is a measurement scale for pain, consisting straight line of 10-cm, the left edge shows “no pain” (0) and the far right edge shows the “worst pain imaginable” (10). The subjects will be asked to draw a straight line illustrating the severity of the pain felt during the assessment.

### Follow up

All patients will be followed up at six weeks after rehabilitation and follow-up record forms will be completed. The time of the last rehabilitation training session will be recorded. Electronic follow-up rehabilitation records will be preserved. When patients drop out of the trial, the reasons for withdrawal will be recorded in detail. Comprehensive and supportive patient communication will be undertaken; patients lost to follow-up because of any reason will be got in touch as soon as possible and be followed up within six weeks. Data regarding patients withdrawal from the study will be included in the final analysis, according to the intention-to-treat analysis principle.

### Data management

Data from the trial will be kept in a secure, locked storage area with limited access for later review by a biostatistician, a researcher in charge.

### Power analysis

The primary outcome measure will be a DASH score in the study with the minimal clinically important difference (MCID) of 10.83 points and standard deviation (SD) of 12.85 points [20]. In power calculations, we calculated the sample size required per group to be 20 patients with 95% confidence interval, power of 0.95 and effect size of 0.8. Therefore 20 patients in both groups have to complete 6 weeks of follow-up to have adequate statistical power.

### Statistical analysis

The SPSS (27.0) will be used to perform statistical analysis. To compare the group effect, two-way repeated measures analysis of variance (ANOVA) will be used. If subjects are lost to follow up, an intention-to-treat analysis will be carried out. When ANOVA reveals a significant difference, student’s t test will be used to compare the changes within the group. Statistical significance is determined by a two-sided p value of less than 0.05. Mann-Whitney U, t-test or Fisher’s exact test will be used for comparing Groups at baseline. Mann-Whitney U test will be used to compare primary and secondary outcomes between the groups at 6 weeks.

## Discussion

The goal of this experimental study is to evaluate the impact of leap motion control after DRF on functional independence. The leap motion tracking device has proved its efficacy in terms for improving the dexterity of hand following exergaming in patients suffering from Parkinsonism [20]. It has also shown its effectiveness in the patients with spinal cord injury presenting with upper limb limited skills [21]. Nonetheless, it was proposed that leap motion control may have more positive effects in enhancing patient’s hand function after distal radius fracture as it provides an improved motion control, extra spatial transformation and more patient entertainment to encourage motion learning. The findings of this research may or may not provide evidence supporting this hypothesis.

To conclude, this research seeks to examine the rapid and long term effects of leap motion control in DRF patients. The study findings would help prospective patients with DRF, which may include a newly designed method of rehabilitation. A number of related articles in this region were reviewed.

## Supporting information

S1 File (PDF)

S1 Checklist (DOCX)

S1 Data (DOCX)

S1 Appendix (DOCX)
