# Supplementary material for: A research protocol on leap motion tracking device: A novel intervention method in distal radial fracture rehabilitation
Source: PLoS One. 2022 May 6;17(5):e0267549. doi: 10.1371/journal.pone.0267549 (PMC9075655; doi:10.1371/journal.pone.0267549)
Supplement: S1 File — (PDF) [file pone.0267549.s001.pdf]

Clinical Trial Details (PDF Generation Date :- Sat, 22 May 2021 05:28:25 GMT)

**CTRI Number**	CTRI/2021/05/033498 [Registered on: 10/05/2021] - **Trial Registered Prospectively**
**Last Modified On**	08/05/2021
**Post Graduate Thesis**	Yes
**Type of Trial**	Interventional
**Type of Study**	Physiotherapy (Not Including YOGA)
**Study Design**	Randomized, Parallel Group Trial
**Public Title of Study**	Effect of Leap Motion Tracking Device on Pain, Range of Motion, Muscle Strength And Functional Parameters in patients with Distal Radial Fracture
**Scientific Title of Study**	Efficacy of Leap Motion Tracking Device versus Conventional Rehabilitation on Pain, Range of Motion, Muscle Strength and Functional Parameters in patients with Distal Radial Fracture

Secondary IDs if Any

Secondary ID	Identifier
NIL	NIL

Details of Principal Investigator or overall Trial Coordinator (multi-center study)

Details of Principal Investigator	
**Name**	Sakshi P Arora
**Designation**	MPT Student
**Affiliation**	Ravi Nair Physiotherapy College
**Address**	Department of Community Health Physiotherapy, Ravi Nair Physiotherapy College, Datta Meghe Institute of Medical Sciences, Sawangi(M), Wardha, Maharashtra, India.<br>Wardha<br>MAHARASHTRA<br>442001<br>India
**Phone**	9422889418
**Fax**	
**Email**	sakshiparora@gmail.com

Details Contact Person (Scientific Query)

Details Contact Person (Scientific Query)	
**Name**	Dr Waqar M Naqvi
**Designation**	Professor and Head of Department
**Affiliation**	Ravi Nair Physiotherapy College
**Address**	Department of Community Health Physiotherapy, Ravi Nair Physiotherapy College, Datta Meghe Institute of Medical Sciences, Sawangi (M), Wardha, Maharashtra, India.<br>Wardha<br>MAHARASHTRA<br>442001<br>India
**Phone**	9021699000
**Fax**	
**Email**	waqar.naqvi@dmimsu.edu.in

Details Contact Person (Public Query)

Details Contact Person (Public Query)	
**Name**	Dr Waqar M Naqvi
**Designation**	Professor and Head of Department
**Affiliation**	Ravi Nair Physiotherapy College
**Address**	Department of Community Health Physiotherapy, Ravi Nair Physiotherapy College, Datta Meghe Institute of Medical Sciences, Sawangi (M), Wardha, Maharashtra, India.<br>Wardha<br>MAHARASHTRA

		442001<br>India
	Phone	9021699000
	Fax	
	Email	waqar.naqvi@dmimsu.edu.in

Source of Monetary or Material Support

Source of Monetary or Material Support
> RNPC, AVBRH and Datta Meghe Institute of Medical Sciences, Sawangi, Meghe, Wardha, Maharashtra, India.

Primary Sponsor

Primary Sponsor Details	
Name	Datta Meghe Institute of Medical Sciences Ravi Nair Physiotherapy College
Address	Department of Community Health Physiotherapy, Ravi Nair Physiotherapy College, Datta Meghe Institute of Medical Sciences, Sawangi, Meghe, Wardha, Maharashtra, India.
Type of Sponsor	Private medical college

Details of Secondary Sponsor

Name	Address
NIL	NIL

Countries of Recruitment

List of Countries
India

Sites of Study

Name of Principal Investigator	Name of Site	Site Address	Phone/Fax/Email
Sakshi P Arora	Ravi Nair Physiotherapy OPD, Acharya Vinoba Bhave Rural Hospital.	Department of Community Health Physiotherapy, Ravi Nair Physiotherapy College, Datta Meghe Institute of Medical Sciences, Sawangi(M), Wardha, Maharashtra, India.<br>Wardha<br>MAHARASHTRA	9422889418<br><br>sakshiparora@gmail.com

Details of Ethics Committee

Name of Committee	Approval Status	Date of Approval	Is Independent Ethics Committee?
Departmental Research Committee, RNPC	Approved	31/03/2021	No

Regulatory Clearance Status from DCGI

Status	Date
Not Applicable	No Date Specified

Health Condition / Problems Studied

Health Type	Condition
Patients	Fracture at wrist and hand level

Intervention / Comparator Agent

Type	Name	Details
Comparator Agent	Conventional Physiotherapy Treatment	Whirlpool bath, Maitland Mobilization grade I and II for pain management, Stretching, Strengthening with Theraband for 60 minutes.
Intervention	Leap Motion Controller Device	5 games, each game concentrating on the specific wrist movement, for 5 minutes each, for 30 minutes of the treatment session.

Inclusion Criteria

Inclusion Criteria	
**Age From**	18.00 Year(s)
**Age To**	50.00 Year(s)
**Gender**	Both
**Details**	1. Patients must be diagnosed with an A3 extraarticular multi-fragmentary distal radial fracture type and treated conservatively with plaster cast immobilization and closed reduction.<br>2. Participants must accept and sign the Informed consent.<br>3. No previous history of wrist/ hand fracture, history of inflammatory arthritis, or any possible upper limb fracture.<br>4. Patients who are able to comprehend

Exclusion Criteria

Exclusion Criteria	
**Details**	1. Participants who were treated for DRF reduction and/or fixation with some form of surgical intervention (e.g. external fixation, volar plate, and Kirschner wires).<br>2. Patients with Mini-Mental score less than 26 points on the examination.<br>3. After the removal of the immobilization, patients with immediate complications like malunion or non-union.<br>4. Patients with past trauma either in arms or hands that had impaired function.<br>5. Patients with inflammatory or non-inflammatory diseases and neurological disorder were excluded.

**Method of Generating Random Sequence**	Computer generated randomization
**Method of Concealment**	Sequentially numbered, sealed, opaque envelopes
**Blinding/Masking**	Participant Blinded

Primary Outcome

Outcome	Timepoints
1. Disabilities of the Arm, Shoulder and Hand Outcome Questionnaire (DASH)<br>2. Universal Goniometer	1. Disabilities of the Arm, Shoulder and Hand Outcome Questionnaire (DASH)<br>2. Universal Goniometer

Secondary Outcome

Outcome	Timepoints
1. Visual Analogue Scale (VAS)<br>2. Grip Strength using Hand Dynamometer	Pre Treatment day 1<br>Post Treatment 6 weeks

**Target Sample Size**	**Total Sample Size=**40<br>**Sample Size from India=**40<br>**Final Enrollment numbers achieved (Total)=**Applicable only for Completed/Terminated trials<br>**Final Enrollment numbers achieved (India)=**Applicable only for Completed/Terminated trials
**Phase of Trial**	N/A
**Date of First Enrollment (India)**	11/05/2021
**Date of First Enrollment (Global)**	No Date Specified
**Estimated Duration of Trial**	**Years=**1<br>**Months=**0<br>**Days=**0
**Recruitment Status of Trial (Global)**	Not Applicable
**Recruitment Status of Trial (India)**	Not Yet Recruiting
**Publication Details**	
**Brief Summary**	Current Research will be intending to check the efficacy of leap motion controller device over conventional physiotherapy treatment on pain, range of motion, muscle strength and functional

parameters in distal radial fracture patients.
