# Supplementary material for: A research protocol on leap motion tracking device: A novel intervention method in distal radial fracture rehabilitation
Source: PLoS One. 2022 May 6;17(5):e0267549. doi: 10.1371/journal.pone.0267549 (PMC9075655; doi:10.1371/journal.pone.0267549)

# DATTA MEGHE INSTITUTE OF MEDICAL SCIENCES

**[Deemed to be University]**

**Sawangi (Meghe), Wardha, Maharashtra, India**

**DEPARTMENT OF COMMUNITY HEALTH PHYSIOTHERAPY**

**RAVI NAIR PHYSIOTHERAPY COLLEGE**

**SAWANGI (MEGHE), WARDHA.**

**SYNOPSIS**

**TITLE: Efficacy of Leap Motion Tracking Device versus Conventional Rehabilitation on Pain, Range of Motion, Muscle Strength And Functional Parameters in patients with Distal Radial Fracture**

**INVESTIGATOR:**

**Sakshi P. Arora**

First Year Postgraduate Student, Department of Community Health Physiotherapy,

Ravi Nair Physiotherapy College, Sawangi (Meghe), Wardha-442004.

**GUIDE:**

**Prof. Waqar M. Naqvi (PT)**

Professor & Head, Department of Community Health Physiotherapy, Ravi Nair Physiotherapy College, Sawangi (Meghe), Wardha-442004.

**Date of Submission:**

| **Datta Meghe Institute of Medical Sciences**  (Deemed to be University)  **INSTITUTIONAL ETHICS COMMITTEE**  **Proforma for PG Thesis-MD/MS/MDS/M.Sc./Ph.D./M.Phil./Fellowship diploma &**  **All research proposals-projects/synopsis**  **Name of the Institute Ravi Nair Physiotherapy College** **Department of** **Community Health Physiotherapy** | |
| --- | --- |
| Name of the Principal Investigator | Sakshi Arora |
| Name of the Co-investigator/Guide | Prof. Waqar Naqvi (PT) |
| Course of Study and Subject | 2 years |
| Date of Admission | 7^th^ October, 2020 |
| Topic / Title of the Project | Efficacy of Leap motion tracking device versus conventional rehabilitation on pain, range of motion, muscle strength and functional parameters in patients with distal radial fracture |
| SYNOPSIS (as per the guidelines) | Attached |
| Signature of the Candidate |  |
| Mobile No. , e-mail | 9422889418, sakshi.arora@dmimsu.edu.in |
| Name and Designation of the Guide Mobile No. & email | Prof. Waqar Naqvi (PT), Professor & Head, Department of Community Health Physiotherapy, Ravi Nair Physiotherapy College |
| Signature of Guide |  |
| Technical Soundness (rationale, literature review, objective, methodology, references) |  |
| Feasibility (If any other Dept. & Institute is involved name & signature of the head) | Technical:  Infrastructural:  Financial: |
| Remarks & Signature of Chairman Departmental Research Committee |  |

| **Datta Meghe Institute of Medical Sciences**  **(Deemed to be University)**  **INSTITUTIONAL ETHICS COMMITTEE**  **Clearance from Scientific Scrutiny Committee/ Institutional Research Committee** | |
| --- | --- |
| Name of the Principal Investigator | Sakshi Arora |
| Department | Community Health Physiotherapy |
| Name of the Institute | Ravi Nair Physiotherapy College |
| Course of Study and Subject | 2 Years |
| Date of Admission | 7^th^ October, 2020 |
| Title of Project | Efficacy of Leap motion tracking device versus conventional rehabilitation on pain, range of motion, muscle strength and functional parameters in patients with distal radial fracture |
| SYNOPSIS (as per the guidelines) | Attached |
| Signature of the Candidate |  |
| Mobile No. , e-mail | 9422889418, [sakshi.arora@dmimsu.edu.in](mailto:sakshi.arora@dmimsu.edu.in) |
| Name and Designation of the Guide | Prof. Waqar Naqvi (PT), Professor & Head, Department of Community Health Physiotherapy, Ravi Nair Physiotherapy College |
| Signature of Guide |  |
| Technical Soundness (rationale, literature review, objective, methodology, references) |  |
| Feasibility | Technical:  Infrastructural:  Financial : |
| Remarks & Signature of Chairman Scientific Scrutiny Committee |  |

CENTRAL RESEARCH LABORATORY

**Proforma of Feasibility Criteria for Investigations**

**Name of Principle Investigator (PI):** Sakshi P. Arora

**Name of Guide:** Prof. Waqar M. Naqvi (PT)

**Department:** Community Health Physiotherapy

**Title of Project:** Efficacy of Leap Motion Tracking Device versus Conventional Rehabilitation on Pain, Range of Motion, Muscle Strength And Functional Parameters in patients with Distal Radial Fracture

| **Sr.**  **No.** | **Investigation required** | **Quantity** | **Laboratory in which the Investigation will be carried out** | **Signature of Laboratory In-charge** |
| --- | --- | --- | --- | --- |
| 1. |  |  |  |  |
| 2. |  |  |  |  |
| 3. |  |  |  |  |
| 4. |  |  |  |  |
| 5. |  |  |  |  |
| 6. |  |  |  |  |

Signature of PI Signature of Guide

Signature of CRL Representative

**Datta Meghe Institute of Medical Sciences**

# (Deemed to be University)

## INSTITUTIONAL ETHICS COMMITTEE

**Submission of Research Synopsis/Proposal Ethics Review Checklist**

**INDEX**

*[****√*** *please select-Attached]*

## Synopsis along with the forms of the IEC

- Provisional Registration Form for Ph.D. (Doctor of Philosophy) in the

Faculty Medicine/Dentistry/Ayurveda/Nursing/InterdisciplinarySciences/Physiotherapy.

## Proforma of IEC (duly signed by Head of departmental research committee & Scientific Scrutiny Committee Chairman)

- Sample size justification
- Case history proforma (if applicable)
- Key articles
- Informed consent forms (English & Hindi/Marathi)

## Budget disclosure form

**Index**

| **Sr. No.** | **Topic** | **Page No.** |
| --- | --- | --- |
| 1. | Introduction | 7-9 |
| 2. | Aim and Objectives | 9 |
| 3. | Hypothesis | 9-10 |
| 4. | Review Of Literature | 10 |
| 5. | Methodology | 11-15 |
| 6. | Scope of study and Implications | 15 |
| 7. | References | 16-18 |
| 8. | Annexure I- Consent Form | 19 |
| 9. | Annexure II- Questionnaire | 20-23 |

**Introduction:**

Distal radial fractures (DRF) is a frequent site of injury in upper extremity fracture and are amongst the mostly encountered fractures in emergency rooms (Hong et al., 2020). A DRF is typically described by fracture at a junction of the cortical bone where it is thinner and trabecular bone network with a reinforcement of around 2cm distally from the articulating surface of the radial (MacIntyre and Dewan, 2016). There is a considerable association of DRF on functional activities of patient affecting the socioeconomic costs and deteriorating standard of living (Meijer et al., 2019). The mechanism of injury follows a force axially placed across the bone with bone density determining the injury pattern along with the joint position following the magnitude and covering the direction of the force, however, maximum DRFs are the outcome of falls with the wrist extended and pronated. During the mechanism of injury, if a dorsal bending force across the distal radial is being placed then it is referred to as a FOOSH (fall onto an outstretched hand) (Hsu et al., 2020). Being most common upper limb fracture in all age groups with bimodal distribution, the DRF peaks in young men and in post-menopausal women with incidence ratio of 1:4 (Rundgren et al., 2020) (Hsu et al., 2020). Younger patients follow mechanism with higher energy trauma whereas elderly patients with associated osteoporosis follow lower energy falls. Following the variabilities in nature of injury, the distal radial fracture includes multiple classification systems based on pattern of intraarticular involvement as Frykman classification and based on mechanism of injury as Fernandez classification (Hsu et al., 2020). Simple classifications were made based on clinical appearance and often named after those who described them (Karantana et al., 2020). The term Colles’s fracture refers to the DRF involving both intraarticular and extraarticular surfaces with dorsal angulation, displacement and radial shortening, however, Smith fracture is extraarticular DRF with volar angulation. The Barton fracture is the dorsal or volar rim fracture with volar displacement and avulsion fracture of the radial styloid is referred to as Chauffeur’s fracture (Rundgren et al., 2020).

In younger people, DRF is mostly associated with fall associated during sports events and road traffic accidents (Burhani and Naqvi, 2020). The incidence of DRF in females is significantly greater than in males incorporating the menopause as responsible factor leading to osteoporosis associated with reduced bone density showing peak between the age group of 60 and 70 (Gutiérrez-Espinoza et al., 2017).

Considering the injury patterns and patient profiles being heterogenous in nature, the management line should prefer the severity of the injury, the desired functional independency of patients along with existing comorbidities. In older adults, the preferred line of management is conservative or non-operative to have good results with foundation stone of immobilisation. Surgical management options include closed reduction and application of a cast, percutaneous K-wires, open reduction and internal fixation with plates, or external fixation according to the patient requirements (Vaghela et al., 2020). Following DRF, many factors manipulate the recovery of the patient implying the age, gender, site and extent of injury, management line followed to manage the respective injury, compensations, patient’s education regarding the condition, radial shortening, and intra-articular involvement (Björk et al., 2020). Patients generally recover within 3-6 months to maximum range of motion, strength and function whether managed conservatively or surgically.

Patients with DRF after a duration of immobilisation are often referred for physiotherapy. In the clinical setting physiotherapists use disability assessment, such as range of motion and grip strength, to determine progress as well as outcomes. Hand activity requires a combination of adequate sensation, proprioception, intact neurological control and coordination, appropriate anatomical alignment, and muscle strength and flexibility (Gutiérrez-Espinoza et al., 2017). Physiotherapy interventions are techniques used to improve functional recovery following a distal radial fracture (Bruder et al., 2013). Physical therapy (PT) is of critical importance after the immobilization phase. PT is recommended for reducing pain, increasing range of motion (ROM) and enhancing muscle function and muscle strength. Early rehabilitation concentrate on oedema management, pain reduction as well as shoulder and finger motions (Björk et al., 2020). The therapeutic methods applied to attain these goals may be categorized as active or passive methods. Active treatment includes approaches in which patients has to participate actively in their treatment, such as counselling, a home exercise program (HEP), or supervised programme by a physiotherapist (Smith et al., 2004). Passive treatments relate to interventions where the patient plays a passive part during its procedure, such as mobilization of joint (JM), massage and the use of hot pack, TENS and ultrasound (Maciel et al., 2005).

The use of virtual reality (VR) technology platform in a healthcare environment has become increasingly common over the last two decades. This was rooted in clinical practice. The introduction of VR technology into traditional training has the ability to further increase the outcomes of the training. VR allows users to actively interact in real-time with a simulated environment and offers the opportunity to practice skills learned in the virtual environments to everyday life (Huang et al., 2019). VR-based training has the ability to promote implicit learning, improve variety, and involve the patient actively during the training. Such characteristics are crucial in the optimization of motor learning and could maximize the training impact.

The Leap Motion Controller is an infrared light detector developed as a means of hand gesture and motion recognition device. The corresponding software applies algorithms to the sensor data detected from the hands and generates a 3D representation of contour, position and movement. Current applications include gaming, education, maps and navigating the computer desktop. The technology lends itself to monitoring hand movement exercises used for wrist physiotherapy. However, no current research exists that investigates the feasibility and validity of using this technology.

**Aim:** To compare the efficacy of leap motion tracking device based rehabilitation and conventional rehabilitation in distal radial fracture patients.

**Objectives:**

1. To assess the effect of leap motion tracking device on pain in distal radial fracture patients.
2. To examine the effect of leap motion tracking device on range of motion in distal radial fracture patients.
3. To evaluate the effect of leap motion tracking device on muscle strength in distal radial fracture patients.
4. To investigate the effect of leap motion tracking device on functional parameters in distal radial fracture patients.

**Hypothesis:**

- **Null hypothesis:** There is no significant effect of Leap motion tracking device in rehabilitation of distal radial fracture.
- **Alternate hypothesis:** There is significant effect of Leap motion tracking device in rehabilitation of distal radial fracture than conventional physiotherapy, either equal or superseding.

**Review of Literature**:

1. Héctor Gutiérrez-Espinoza et al. performed a randomized controlled trial to assess and analyse the improvement in functional status and pain in patients following the physiotherapy plan with supervision and home exercise program without supervision in distal radial fracture with an age limit of more than 60 years. The results included that as exercise program under physiotherapy supervision was more effective in short term and medium term for functional improvement for the patients with distal radial fracture.
2. Andrea Bruder et al did a systematic review on Exercise reduces impairment and improves activity in people after some upper limb fractures. In this study 13 relevant trials involving 781 participants with an upper limb fracture were identified. It concluded that the specific regimen of exercises play an evident role in decreasing impairments and improving UL function post specific UL fractures.
3. Andrea M. Bruder et al performed an observational study on physiotherapy intervention practice patterns used in rehabilitation after distal radial fracture. The study showed that after DRF, the interventional exercises are prescribed without considering the physiotherapist approach and patient’s need. The management included interventions in restoring mobility of wrist following principles of managing fracture and an approach with self-management.
4. Verica Filipova et al performed a randomized controlled trial evaluating the effectiveness of combination of physiotherapy and occupational therapy as compared to physiotherapy alone in patients with distal radial fracture treated conservatively. However, the combination of therapies showed statistically significant improvement in strength of grip as compared to only physiotherapy after the fracture. The efficacy was not supported with DASH score results.

**Material and Methodology:**

**Study design:** This interventional comparative study will be carried out in the HumEn Research Lab of Ravi Nair Physiotherapy College, Sawangi (Meghe), Wardha, after approval from Institutional Ethics Committee of Datta Meghe Institute of Medical Sciences, Deemed to be University. Before inclusion, all the participants will be informed regarding the aim and procedure of research. Those participants who will meet the inclusion criteria must give the written informed consent. In an experimental study, those participants (N= 40) diagnosed with distal radial fracture will be enrolled for 6 weeks protocol. The participants will be randomly assigned in a 1:1 ratio, a 6-week therapy with leap motion tracking device and a 6-week traditional therapy. Figure 1 shows the flow chart of the study.

**Recruit Subjects(N=40**)

Subjects will be screened by inclusion and exclusion criteria, informed consent & medical history will be obtained from subjects

**Perform baseline assessment**

**Allocation**

**Control Group**

20 subjects

**Experimental Group**

20 subjects

**6 weeks intervention**

Conventional rehabilitation=60 min/day

**6 weeks intervention**

Leap motion control rehabilitation=30 min/day

**Perform post treatment assessment**

**Statistical Analysis**

**Participants:**

Inclusion criteria are as follows:

1. Age 18-50 years, who have been referred from the Dept. of Orthopaedic.
2. Patients must be diagnosed with an A3 extraarticular multi-fragmentary distal radial fracture type and treated conservatively with plaster cast immobilization and closed reduction.
3. Participants must accept and sign the Informed consent.
4. No previous history of wrist/ hand fracture, history of inflammatory arthritis, or any possible upper limb fracture.
5. Patients who are able to comprehend (Lyngcoln et al., 2005).

Exclusion criteria are as follows:

1. Participants who were treated for DRF reduction and/or fixation with some form of surgical intervention (e.g. external fixation, volar plate, and Kirschner wires).
2. Patients with Mini-Mental score less than 26 points on the examination.
3. After the removal of the immobilization, patients with immediate complications like malunion or non-union.
4. Patients with past trauma either in arms or hands that had impaired function.
5. Patients with inflammatory or non-inflammatory diseases and neurological disorder were excluded.

**Sample size consideration:** This is an experimental study with two-group design that examines the effect of leap motion control in distal radial fracture rehabilitation. Analysis of previous power using G*Power is used to determine the same size (Engel et al., 2019). 40 participants will be enrolled to the control group or experimental group and will be allocated accordingly (Maciel et al., 2005). The G*Power analysis followed the following terms for calculation of sample size which is graphical represented in figure 2.

**t tests** - Means: Difference between two independent means (two groups)

**Analysis:** A priori: Compute required sample size

**Input:** Tail(s) = One

Effect size d = 0.8

α err prob = 0.2

Power (1-β err prob) = 0.95

Allocation ratio N2/N1 = 1

**Output:** Non-centrality parameter δ = 2.5298221

Critical t = 0.8511828

Df = 38

Sample size group 1 = 20

Sample size group 2 = 20

Total sample size = 40

Actual power = 0.9531573

**Figure 2:** The calculation of sample size using G*Power analysis

**Intervention design:**

Group A:

- The carefully monitored physiotherapy program will include active exercises of wrist and hand at 34°C in a whirlpool.
- After that joint mobilization will be performed to the radiocarpal joint. Subjects will undergo grade I or II mobilization of Maitland approach during the first 2 weeks with a dosage of 1cps (cycle per second) for 1 minute.
- In the following 4 weeks, the sustained mobilization of Grade III Maitland approach in anteroposterior and posteroanterior directions will be done with the stabilization of distal radius in neutral position.
- The end range of motion will be attained and the treatment will be progressed with mobilization Grade IV technique.
- Strengthening technique will include exercises with theraband. This treatment will be performed for 60 mins per day for 5 days a week for 6 weeks.

Group B: Those subjects in experimental group with the Leap motion control will be asked to actively move their fingers from initial resting position and execute maximally the following five movements with their wrist and hand: fingers flexion and extension, flexion and extension of the thumb, wrist radial and ulnar deviation, forearm pronation and supination and wrist flexion and extension (Fernández-González et al., 2019) for 30 minutes regularly. Rehabilitation games include-

1. Rhythm game: This game is aimed at helping patients perform flexion exercises for wrist joint. The buttons fall from above as music plays in the background, and must be pushed at the right time by the player.
2. Flappy Bird Clone: It is designed for wrist flexion and extension both in which the player is asked to control a bird flying over a series of tubes before the track ends.
3. Skiing Game: It requires wrist movement i.e. either extension or flexion and wrist radial or ulnar deviation. A skier who comes down a slalom track is guided by the player and must cross the gates (Corona et al., 2018).

**Outcome measures:**

Primary outcome measure:

1. Disabilities of the Arm, Shoulder, and Hand Outcome Questionnaire (DASH): It is a 30- item questionnaire that evaluates the patient’s ability to perform upper extremity activities for functionality.
2. Universal goniometer for ROM: Active flexion and extension, radial deviation, ulnar deviation, pronation and supination of the wrist will be measured by a goniometer. Participants will be assessed to expose their arms in a seated position and to remove any accessories (Tremayne et al., 2002).

Secondary outcome measure:

1. Grip strength : To check grip strength a Jamar dynamometer will be used. The participants would be tested in sitting position with the arm and shoulder in neutral position and elbow in 90 degrees of flexion, and neutral forearm (Tremayne et al., 2002). Then, subjects will be asked with all their strength to make tight fists to maintain that position for 4 seconds and then rest for 30 seconds. First, the unaffected side will be tested and then affected side will be tested. The maximum value obtained from 3 trials will be recorded.
2. VAS: The visual analogue scale is a measurement scale for pain, consisting straight line of 10 cm, the left edge shows “no pain” and the right edge shows the “worst pain”. The subjects will be asked to draw a straight line illustrating the severity of the pain felt during the assessment.

**Follow up:** All patients will be followed up at 6 weeks after rehabilitation and follow-up record forms will be completed. The time of the last rehabilitation training session will be recorded. Electronic follow-up rehabilitation records will be preserved. When patients drop out of the trial, the reasons for withdrawal will be recorded in detail. Comprehensive and supportive patient communication will be undertaken; patients lost to follow-up because of any reason will be got in touch as soon as possible and be followed up within 6 weeks. Data regarding patients withdrawal from the study.

**Data management:** Data from the trial will be kept in a secure, locked storage area with limited access for later review by a biostatistician, a researcher in charge.

**Power analysis:** With G*power analysis, the sample size in respective group is calculated to be 20 patients with 95% confidence interval, power of 0.95 and effect size of 0.8. Therefore 20 patients in both groups have to complete 6 weeks of follow-up to have adequate statistical power.

**Statistical analysis:** The SPSS latest version will be used to perform statistical analysis. To compare the group effect, two-way repeated measures ANOVA (analysis of variance) will be used. When ANOVA reveals a significant difference, student’s t test will be used to compare the changes within the group. Statistical significance is determined by a two-sided p value of less than 0.05. Mann-Whitney U, t-test or Fisher’s exact test will be used for comparing Groups at baseline and for comparing the primary and secondary outcomes between the groups at 6 weeks.

**Scope:**

Leap motion tracking device is an innovative technique which may be used in initial period of rehabilitation for early recovery in distal radial fracture. Also, the studies concentrating on the effectiveness of Leap motion tracking device is limited making it difficult to conclude as an intervention in DRF. Hence, it is necessary to conduct a new experimental study with more standardization in type of intervention and treatment protocol along with larger samples focusing on the effectiveness of leap motion tracking device.

**Implication:** In the present study it can be implied that, Leap motion tracking device based therapy can be an important technique used regularly for the patients with distal radial fracture.

**References:**

1. Björk, M., Niklasson, J., Westerdahl, E., Sagerfors, M., 2020. Self-efficacy corresponds to wrist function after combined plating of distal radial fractures. J. Hand Ther. Off. J. Am. Soc. Hand Ther. 33, 314–319. https://doi.org/10.1016/j.jht.2020.01.001
2. Bruder, A.M., Taylor, N.F., Dodd, K.J., Shields, N., 2013. Physiotherapy intervention practice patterns used in rehabilitation after distal radial fracture. Physiotherapy 99, 233–240. https://doi.org/10.1016/j.physio.2012.09.003
3. Corona, F., Chiuri, R.M., Filocamo, G., Foa’, M., Lanzi, P.L., Lopopolo, A., Petaccia, A., 2018. Serious Games for Wrist Rehabilitation in Juvenile Idiopathic Arthritis. ArXiv180500901 Cs.
4. Engel, Y., Noordijk, S., Spoelder, A., van Gelderen, M., 2019. Self-Compassion When Coping With Venture Obstacles: Loving-Kindness Meditation and Entrepreneurial Fear of Failure. Entrep. Theory Pract. 1042258719890991. https://doi.org/10.1177/1042258719890991
5. Fernández-González, P., Carratalá-Tejada, M., Monge-Pereira, E., Collado-Vázquez, S., Sánchez-Herrera Baeza, P., Cuesta-Gómez, A., Oña-Simbaña, E.D., Jardón-Huete, A., Molina-Rueda, F., Balaguer-Bernaldo de Quirós, C., Miangolarra-Page, J.C., Cano-de la Cuerda, R., 2019. Leap motion controlled video game-based therapy for upper limb rehabilitation in patients with Parkinson’s disease: a feasibility study. J. NeuroEngineering Rehabil. 16, 133. https://doi.org/10.1186/s12984-019-0593-x
6. Gutiérrez-Espinoza, H., Rubio-Oyarzún, D., Olguín-Huerta, C., Gutiérrez-Monclus, R., Pinto-Concha, S., Gana-Hervias, G., 2017. Supervised physical therapy vs home exercise program for patients with distal radial fracture: A single-blind randomized clinical study. J. Hand Ther. Off. J. Am. Soc. Hand Ther. 30, 242–252. https://doi.org/10.1016/j.jht.2017.02.001
7. Hong, I.-T., Lee, J.-K., Ha, C., Jo, S., Wang, P.W., Han, S.-H., 2020. Differences in patient and injury characteristics between sports- and non-sports related distal radial fractures. Orthop. Traumatol. Surg. Res. OTSR. https://doi.org/10.1016/j.otsr.2020.06.021
8. Hsu, H., Fahrenkopf, M.P., Nallamothu, S.V., 2020. Wrist Fracture, in: StatPearls. StatPearls Publishing, Treasure Island (FL).
9. Huang, Q., Wu, W., Chen, X., Wu, B., Wu, L., Huang, X., Jiang, S., Huang, L., 2019. Evaluating the effect and mechanism of upper limb motor function recovery induced by immersive virtual-reality-based rehabilitation for subacute stroke subjects: study protocol for a randomized controlled trial. Trials 20, 104. https://doi.org/10.1186/s13063-019-3177-y
10. IMPACT OF IMMERSIVE VIRTUAL REALITY-BASED REHABILITATION ON FUNCTIONAL INDEPENDENCE AND HEALTH RELATED QUALITY OF LIFE AFTER DISTAL RADIAL FRACTURE: A STUDY PROTOCOL FOR A SINGLE BLINDED RANDOMIZED CONTROL TRIAL., 2020. . J. Crit. Rev. 7. https://doi.org/10.31838/jcr.07.09.111
11. Karantana, A., Handoll, H.H., Sabouni, A., 2020. Percutaneous pinning for treating distal radial fractures in adults. Cochrane Database Syst. Rev. 2, CD006080. https://doi.org/10.1002/14651858.CD006080.pub3
12. Lyngcoln, A., Taylor, N., Pizzari, T., Baskus, K., 2005. The relationship between adherence to hand therapy and short-term outcome after distal radial fracture. J. Hand Ther. Off. J. Am. Soc. Hand Ther. 18, 2–8; quiz 9. https://doi.org/10.1197/j.jht.2004.10.008
13. Maciel, J.S., Taylor, N.F., McIlveen, C., 2005. A randomised clinical trial of activity-focussed physiotherapy on patients with distal radial fractures. Arch. Orthop. Trauma Surg. 125, 515–520. https://doi.org/10.1007/s00402-005-0037-x
14. MacIntyre, N.J., Dewan, N., 2016. Epidemiology of distal radial fractures and factors predicting risk and prognosis. J. Hand Ther. Off. J. Am. Soc. Hand Ther. 29, 136–145. https://doi.org/10.1016/j.jht.2016.03.003
15. Magnus, C.R.A., Arnold, C.M., Johnston, G., Dal-Bello Haas, V., Basran, J., Krentz, J.R., Farthing, J.P., 2013. Cross-education for improving strength and mobility after distal radial fractures: a randomized controlled trial. Arch. Phys. Med. Rehabil. 94, 1247–1255. https://doi.org/10.1016/j.apmr.2013.03.005
16. Meijer, H.A.W., Graafland, M., Obdeijn, M.C., Goslings, J.C., Schijven, M.P., 2019. Face Validity and Content Validity of a Game for Distal Radial Fracture Rehabilitation. J. Wrist Surg. 8, 388–394. https://doi.org/10.1055/s-0039-1688948
17. Rundgren, J., Bojan, A., Mellstrand Navarro, C., Enocson, A., 2020. Epidemiology, classification, treatment and mortality of distal radial fractures in adults: an observational study of 23,394 fractures from the national Swedish fracture register. BMC Musculoskelet. Disord. 21, 88. https://doi.org/10.1186/s12891-020-3097-8
18. Tremayne, A., Taylor, N., McBurney, H., Baskus, K., 2002. Correlation of impairment and activity limitation after wrist fracture. Physiother. Res. Int. J. Res. Clin. Phys. Ther. 7, 90–99. https://doi.org/10.1002/pri.245
19. Vaghela, K.R., Velazquez-Pimentel, D., Ahluwalia, A.K., Choraria, A., Hunter, A., 2020. Distal radial fractures: an evidence-based approach to assessment and management. Br. J. Hosp. Med. Lond. Engl. 2005 81, 1–8. https://doi.org/10.12968/hmed.2020.0006
20. Walenkamp, M.M.J., de Muinck Keizer, R.-J., Goslings, J.C., Vos, L.M., Rosenwasser, M.P., Schep, N.W.L., 2015. The Minimum Clinically Important Difference of the Patient-rated Wrist Evaluation Score for Patients With Distal Radial Fractures. Clin. Orthop. 473, 3235–3241. https://doi.org/10.1007/s11999-015-4376-9

**ANNEXURE 1**

**CONSENT FORM**

**Efficacy of Leap motion tracking device versus conventional rehabilitation on pain, range of motion, muscle strength and functional parameters in patients with distal radial fracture**

This is to certify that I………………………………………………………………….have been given that required information with respect to my participation as a volunteer in the above mentioned study. The contents of form have been explained to me in my own language.

I confirm that I will receive a signed copy of consent form. I have understood the nature of the study and I volunteer to participate in this research study as subject.

Name: -…………………………………………………………….

Age/Gender:

Address:-

Contact No.

Date: / /20 Sign: -

Place: Sawangi

I undersigned Dr. Sakshi P. Arora (PT) have explained the study details and have cleared all the quires put forth by above volunteer to the best of my ability. I confirm that all data and test result achieved will be kept strictly confidential and will be withheld from any misuse.

Date: / /20 Sign: -

Place: Sawangi

**Annexures- II**

DASH QUESTIONNAIRE

Please rate your ability to do the following activities in the last week by circling the number below the appropriate response.


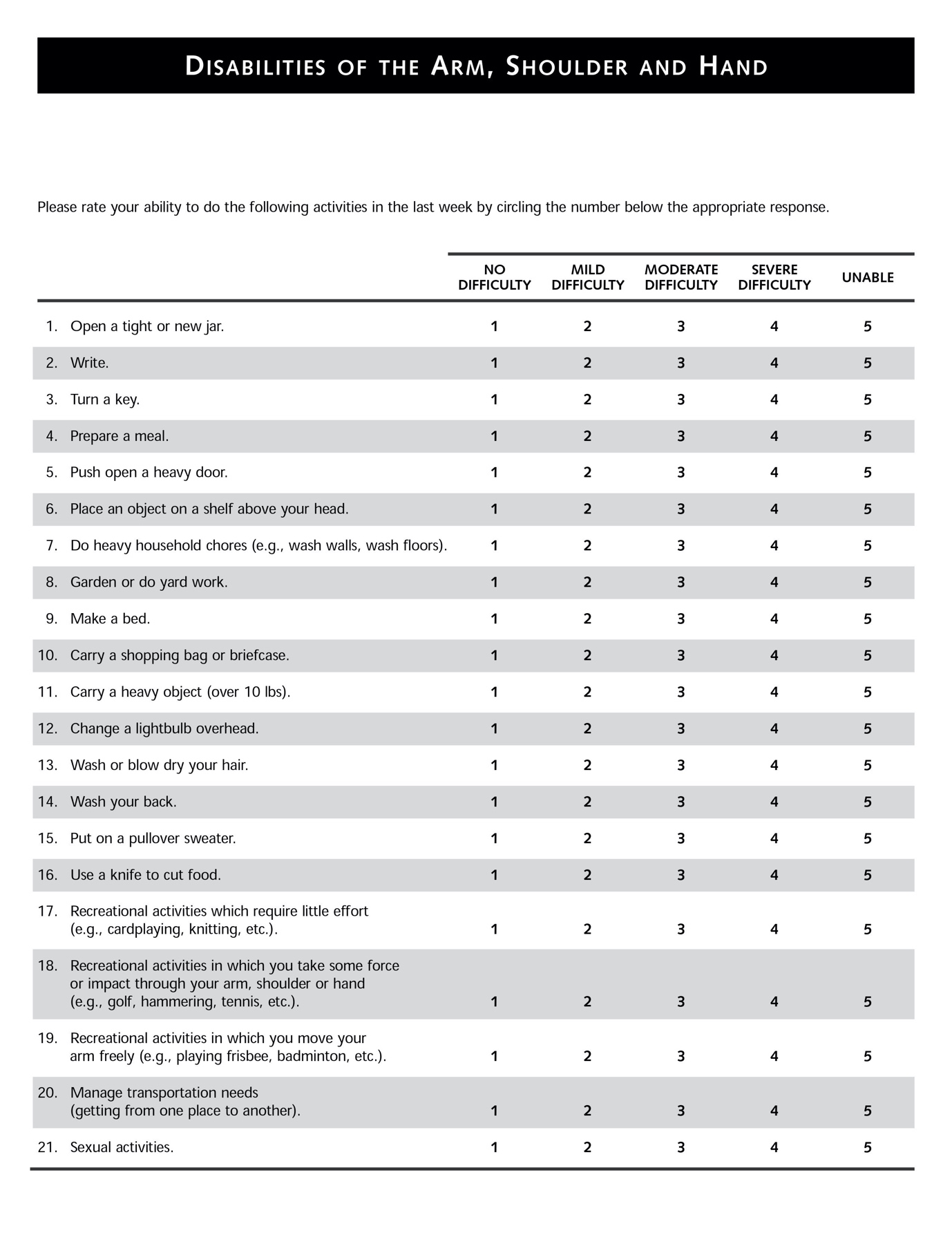


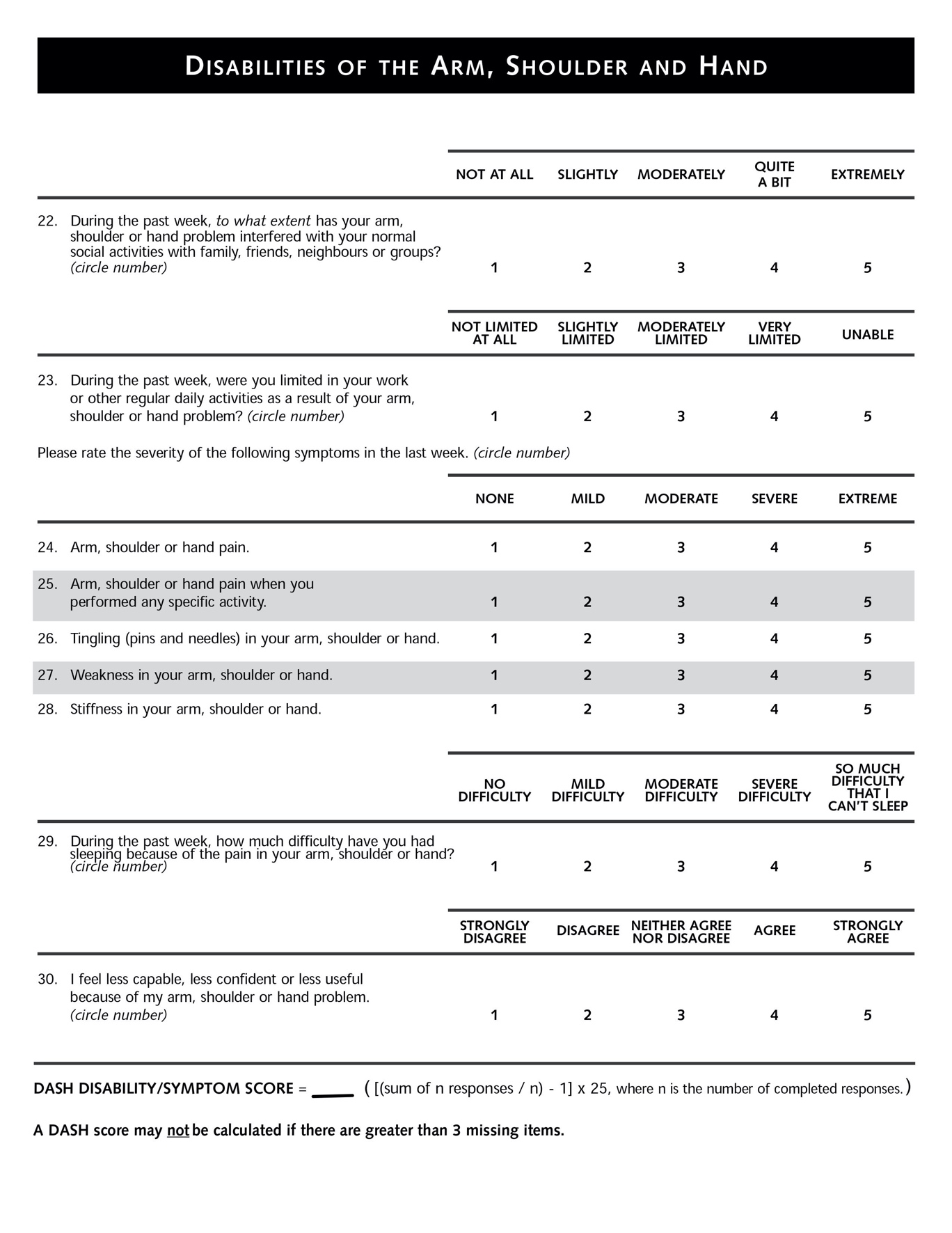

Supplement: S1 Data — (DOCX) [file pone.0267549.s003.docx]
