# Supplementary material for: A research protocol on leap motion tracking device: A novel intervention method in distal radial fracture rehabilitation
Source: PLoS One. 2022 May 6;17(5):e0267549. doi: 10.1371/journal.pone.0267549 (PMC9075655; doi:10.1371/journal.pone.0267549)
Supplement: S1 Appendix — (DOCX) [file pone.0267549.s004.docx]

**Appendices**

**Consent Form**

**Efficacy of Leap motion tracking device versus conventional rehabilitation on pain, range of motion, muscle strength and functional parameters in patients with distal radial fracture**

This is to certify that I………………………………………………………………….have been given that required information with respect to my participation as a volunteer in the above mentioned study. The contents of form have been explained to me in my own language.

I confirm that I will receive a signed copy of consent form. I have understood the nature of the study and I volunteer to participate in this research study as subject.

Name: -…………………………………………………………….

Age/Gender:

Address:-

Contact No.

Date: / /20 Signature:

Place: Sawangi

I undersigned Dr. Sakshi P. Arora (PT) have explained the study details and have cleared all the queries put forth by above volunteer to the best of my ability. I confirm that all data and test result achieved will be kept strictly confidential and will be withheld from any misuse.

Date: / /20 Signature:

Place: Sawangi

**Data Collection Sheet**

**Assessment Proforma: Group A/ Group B**

Name:

Age: Gender:

DASH Score: Grip Strength:

VAS:

At rest:

Worst pain imaginable

No pain

During activity:

Worst pain imaginable

No pain

Range of motion:

| Shoulder | Right | | Left | |
| --- | --- | --- | --- | --- |
| Flexion |  |  |  |  |
| Extension |  |  |  |  |
| Abduction |  |  |  |  |
| Adduction |  |  |  |  |
| Medial Rotation |  |  |  |  |
| Lateral Rotation |  |  |  |  |

| Elbow | Right | | Left | |
| --- | --- | --- | --- | --- |
| Flexion |  |  |  |  |
| Extension |  |  |  |  |

| Wrist | Right | | Left | |
| --- | --- | --- | --- | --- |
| Flexion |  |  |  |  |
| Extension |  |  |  |  |
| Radial Deviation |  |  |  |  |
| Ulnar Deviation |  |  |  |  |
| Supination |  |  |  |  |
| Pronation |  |  |  |  |
